# Maximal Fat Oxidation During Exercise in Healthy Individuals: Lack of Genetic Association with the *FTO* rs9939609 Polymorphism

**DOI:** 10.3390/genes16010004

**Published:** 2024-12-24

**Authors:** Teresa García-Pastor, Iván Muñoz-Puente, Miriam Pérez-Pelayo, Isabel Púa, Justin D. Roberts, Juan Del Coso

**Affiliations:** 1 Exercise Physiology Laboratory (GIDECS), Facultad HM de Ciencias de la Salud, Universidad Camilo José Cela, 28692 Villanueva de la Cañada, Madrid, Spain; ivan@ivientrenadorpersonal.com; 2 Instituto de Investigación Sanitaria HM Hospitales, 28692 Madrid, Spain; 3 Severo Ochoa Hospital, 28914 Leganés, Madrid, Spain; mppelayo@salud.madrid.org (M.P.-P.); isabel.pua@salud.madrid.org (I.P.); 4 Cambridge Centre for Sport and Exercise Sciences, Faculty of Science and Engineering, Anglia Ruskin University, Cambridge CB1 1PT, UK; justin.roberts@aru.ac.uk; 5 Sport Sciences Research Centre, Rey Juan Carlos University, 28943 Fuenlabrada, Madrid, Spain

**Keywords:** genetic variation, overweight, lipid oxidation, genetic association studies, fat accumulation

## Abstract

**Background/Objectives**: Previous studies suggest that there is a genetically determined component of fat oxidation at rest and during exercise. To date, the *FTO* gene has been proposed as a candidate gene to affect fat oxidation during exercise because of the association of the “at-risk” A allele with different obesity-related factors such as increased body fat, higher appetite and elevated insulin and triglyceride levels. The A allele of the *FTO* gene may also be linked to obesity through a reduced capacity for fat oxidation during exercise, a topic that remains largely underexplored in the current literature. The aim of this study was to analyze the association between the *FTO* rs9939609 polymorphism with the rate of fat oxidation during exercise and metabolic syndrome criteria in healthy participants. **Methods**: A total of 80 healthy participants (41 men and 39 women) underwent comprehensive assessments, including measurements of anthropometric variables, blood pressure and blood measures of fasting glucose, triglycerides, low- and high-density lipoprotein cholesterol (LDL-c and HDL-c), insulin, interleukin-6 (IL-6) and C-reactive protein (CRP) concentrations. Additionally, the Homeostatic Model Assessment (HOMA-IR) was used to evaluate insulin resistance. Peak oxygen uptake (VO_2peak_) and maximal fat oxidation rate (MFO) were also measured during an incremental cycling test. *FTO* rs9939609 genotyping (TT, AT, AA) was performed using genomic DNA samples obtained from a buccal swab and measured with PCR. **Results**: There were 32 participants (40.0%) with the TT genotype; 31 (38.8%) with the AT genotype; and 17 (21.2%) with the AA genotype. Age, body characteristics, VO_2peak_, blood pressure and blood variables were similar across all three genotypes. However, serum insulin concentration and HOMA-IR were associated with the *FTO* rs9939609 genotype with higher values in AA with respect to AT and TT participants (*p* < 0.050). Still, MFO was similar in TT, AT and AA participants (0.35 ± 0.13, 0.37 ± 0.11, 0.33 ± 0.11 g/min, *p* = 0.702). In the dominant model, there was no statistical difference between TT and A allele carriers. However, the recessive model revealed that AA participants had higher values of body mass, body mass index, blood insulin concentration and HOMA-IR than T allele carriers (*p* < 0.050), with no differences in MFO. **Conclusions**: In our sample of healthy individuals, the *FTO* rs9939609 polymorphism was associated with several phenotypes associated with obesity and insulin resistance, particularly under the AA vs. T allele/recessive model. However, the *FTO* rs9939609 polymorphism was not associated with MFO during exercise as fat oxidation was similar across genotypes. This suggests that reduced fat oxidation during exercise is unlikely to be a cause of the obesogenic influence of the *FTO* AA genotype. Clinically, these findings suggest that the obesogenic effects of the *FTO* AA genotype are unlikely driven by impaired fat oxidation during exercise. Instead, attention should focus on mechanisms like appetite regulation and energy intake. Moreover, exercise interventions may still effectively mitigate obesity risk, as AA individuals retain normal fat oxidation capacity during exercise.

## 1. Introduction

Obesity and its associated comorbidities, such as cardiovascular diseases, type 2 diabetes and certain cancers, represent a significant global health challenge [1]. Obesity is characterized by a pathophysiological profile linked to increased morbidity and mortality primarily due to its association with a heightened risk of developing other health conditions [2]. Today, obesity is recognized as a chronic disease with a multifactorial etiology, stemming from genetic predisposition but significantly influenced and modulated by environmental factors [3]. Although excessive body weight and fat accumulation result from an imbalance between energy intake and energy expenditure, the underlying factors and mechanisms contributing to this condition are complex and not yet fully understood [4].

A positive fat balance and gradual weight gain over time may result from an impaired ability to effectively adjust fat oxidation in response to fat intake [5]. Under basal conditions, obese individuals have been shown to exhibit lower fat oxidation levels compared to age-matched controls [6]. Nonetheless, evidence regarding fat oxidation during exercise remains inconsistent and controversial, with studies reporting varying results depending on exercise intensity, duration and individual metabolic characteristics, with some variability added due to the reliability of the methods used to assess fat oxidation.

For example, the maximal fat oxidation rate during exercise (MFO) and the exercise intensity that elicits MFO (Fatmax) have been reported to be lower in obese individuals compared to their lean counterparts [7,8]. However, other studies suggest that MFO and Fatmax can be similar or even higher in obese individuals compared to those of normal weight, likely due to differences in muscle metabolism, substrate availability and measurement protocols [9]. Still, these studies do not clarify whether the potentially reduced MFO and Fatmax observed in obese individuals are contributing factors to the development of obesity or merely consequences of the condition. Further research is needed to establish the directionality of this relationship and the underlying mechanisms involved, such as the role of genetics in fat oxidation. From a clinical perspective, individuals with a genetically induced impairment in their capacity to oxidize fat during exercise may face challenges in meeting energy demands efficiently, resulting in a greater reliance on carbohydrate metabolism and subsequent fat storage. This metabolic imbalance could drive an increased risk of body adiposity and related comorbidities, such as insulin resistance, type 2 diabetes and cardiovascular diseases. Understanding this impaired fat oxidation as a mechanism underlying the impact of certain genetic variants highlights the importance of integrating genetic insights into clinical strategies for obesity prevention and management. It underscores the need for tailored interventions, including personalized exercise and dietary programs, to address these metabolic vulnerabilities and improve health outcomes in genetically predisposed individuals.

Numerous genes are implicated in the pathogenesis of obesity and its associated metabolic impairments. The heterogeneity of the obese phenotype suggests that the capacity for fat oxidation, both at rest and during exercise, may be influenced by genetic predisposition, contributing to individual variability in metabolic responses and susceptibility to obesity [10]. In line with this notion, fat oxidation capacity during exercise may have a genetically determined component, which could predispose individuals to obesity due to a reduced ability to oxidize fat efficiently [11]. Among the genes associated with obesity, the *FTO* (fat mass and obesity-associated) gene was the first robust locus identified through genome-wide association studies (GWASs) to show a significant association with body mass index (BMI) and body fat percentage [12]. This discovery has been consistently replicated, highlighting the *FTO* gene’s critical role in obesity predisposition and related metabolic traits [13]. Specifically for the T>A rs9939609 polymorphism of the *FTO* gene (with three possible genotypes, TT, AT and AA), each copy of the minor A allele is associated with a 31% increase in obesity risk and an average higher body weight of approximately 1.2 kg [12,14]. Individuals with the AA genotype of the *FTO* rs9939609 polymorphism habitually exhibit higher body mass index (BMI) and fat mass compared to AT and TT individuals [15]. However, the underlying mechanisms by which this genetic variation promotes obesity remain unclear and require further investigation.

Interestingly, not all individuals with the AA genotype in the *FTO* rs9939609 polymorphism are overweight/obese, suggesting potential interactions of the genetic predisposition to weight gain/fat accumulation with other biological or environmental factors [3]. For instance, previous studies have highlighted the attenuating effects of physical activity on the association between the *FTO* polymorphism and obesity risk [16]. Additionally, determinants such as cardiorespiratory fitness, sex, age and muscle mass significantly influence maximal fat oxidation (MFO) during exercise [8]. This is significant because the ability to oxidize fat during exercise has been suggested as a protective factor against various cardiometabolic conditions, including insulin resistance and obesity [17]. To date, the association between the *FTO* rs9939609 polymorphism and fat oxidation during exercise remains poorly understood, with limited evidence available. To the authors’ knowledge, only one study has tested the influence of the *FTO* rs9939609 polymorphism on MFO and Fatmax [11]. These authors reported that MFO was higher in the TT participants compared to the AT individuals. However, in this study [11], individuals with the AA genotype exhibited comparable MFO values to those with the TT genotype, which contradicts the typical model of the *FTO* rs9939609 polymorphism, where AA carriers are generally associated with greater obesity-related factors compared to TT carriers. The aim of this study was to examine the association between the *FTO* rs9939609 polymorphism and the rate of fat oxidation during exercise, as well as its relationship with metabolic syndrome criteria in healthy participants. By focusing on healthy individuals, the study sought to determine whether reduced maximal fat oxidation linked to the *FTO* rs9939609 polymorphism contributes to genetic susceptibility to body fat accumulation, even in individuals who have not yet developed obesity or related comorbidities. We hypothesized that AA participants would have lower values of MFO and Fatmax than TT participants, based on evidence previously introduced linking the A allele of the *FTO* rs9939609 polymorphism to obesity-related phenotypes, which could be associated with a diminished capacity to oxidize fat during exercise.

## 2. Materials and Methods

### 2.1. Participants

A total of 80 healthy volunteers (41 men and 39 women) participated in the study. All participants were of European/Caucasian descent. The criteria for inclusion in the study were age between 18 and 48 years, the absence of any diagnosed chronic pathology and overall good health assessed with the PAR-Q+ [18]. The criteria for exclusion were any musculoskeletal injury in the three months preceding the assessment, taking prescribed medications or nutritional supplements (including oral contraceptives) during the month prior to the investigation and smoking. Women were always tested in their luteal phase. Participants’ characteristics, including age, anthropometric characteristics, main metabolic syndrome markers and maximal/peak values at the end of a maximal cycle ergometer test, used to assess peak oxygen uptake (VO_2peak_) are depicted in Table 1. The study protocol was ethically approved by Camilo José Cela University Review Board (013-2016) and was carried out in accordance with the latest version of the Declaration of Helsinki. Before participating in the study, all the participants received complete information including associated study risks/benefits, after which, written informed consent was obtained. As compensation, participants received a report with all their data.

### 2.2. Sample Size Calculation

An a priori sample size calculation was performed by using the G*Power software (v.3.1.9.7, Düsseldorf, Germany) and data from a previous investigation on the association of the *FTO* rs9939609 polymorphism with the fat oxidation rate during exercise [11]. The sample size was calculated to obtain an effect size of 0.8 Cohen’s d units with a statistical power of 0.80 and a two-tailed α level of 0.05 for an analysis of variance (ANOVA; one-way) (three groups, one measurement). This calculation indicated that at least 21 participants were required to obtain statistically significant differences among genotypes. We recruited 80 participants, taking into account the possibility that some of the genotypes may be present in a frequency lower than 33% in the recruited sample.

### 2.3. Experimental Design

A cross-sectional multivariate experimental design was used in order to explain the association of the *FTO* rs9939609 polymorphism with the rate of fat oxidation during exercise and metabolic syndrome criteria. All participants underwent two assessment sessions, separated by less than 7 days. In the first one, blood samples, resting variables (i.e., blood pressure) and anthropometric data were obtained. In the second session, participants performed a maximal incremental test on a cycle ergometer to assess the maximal rate of fat oxidation (MFO). Fatmax was established as the % of VO_2peak_ at which MFO occurred. Ambient temperature and humidity during the experimental testing were controlled to avoid the effect of ambient temperature on fat oxidation during exercise [19]. Both ambient temperature and humidity were recorded at the beginning and the end of each trial (OH1001, OH Haus, Madrid, Spain); they were, on average for all trials, 21.0 ± 0.8 °C and 41.5 ± 5.4%, respectively. All experimental trials were performed in the morning to avoid the influence of circadian rhythm on fat oxidation rates during exercise [20].

### 2.4. Experimental Protocol

On the first day of measurement, participants arrived at the laboratory in the early morning (between 07:00 and 08:00) after they had complied with diet (fasting for 8 h) and exercise (avoiding intense exercise from 24 h before the test) standardizations. After a 10 min resting period seated in a chair, blood pressure was measured in triplicate (M6 Comfort device, Omron Healthcare, Kyoto, Japan). The mean value of these three measurements was used for analysis, and mean arterial pressure was calculated as diastolic blood pressure + 0.33 × (systolic blood pressure − diastolic blood pressure) [21]. After that, a venous blood sample was obtained from an antecubital vein. A portion of each blood sample was introduced in situ into a blood glucose analyzer (Accu-chek, Roche, Basel, Switzerland). The blood sample was allowed to clot for 10 min and was then centrifuged at 5000× *g* for 10 min to obtain serum samples. The serum samples were introduced in clean tubes and frozen at −80 °C until they were analyzed. The serum portion was analyzed for triglycerides, low-density lipoprotein (LDL), high-density lipoprotein (HDL), insulin, interleukin 6 (IL-6) and reactive C-reactive protein (CRP). These variables were analyzed using standardized biochemical processes (enzymatic colorimetric assays for triglycerides, LDL and HDL, chemiluminescent immunoassay for insulin, high-sensitivity immunoassays for IL-6 and high-sensitivity CRP (hs-CRP) immunoturbidimetric assays for CRP) in an accredited laboratory according to ISO17025, which is an international standard specifying requirements for quality and competence in testing and calibration laboratories created by the International Organization for Standardization (ISO). Later, the Homeostatic Model Assessment for insulin resistance (HOMA-IR) was calculated to evaluate insulin resistance based on fasting blood glucose and fasting insulin concentrations, using the following formula: fasting insulin (in μU/mL) × fasting glucose (in mmoL/L)/22.5 [22].

After the blood sample was obtained, a genomic DNA sample was collected via a buccal swab. At a later stage, genomic DNA was isolated from each sample using standardized protocols [23] and genotyping was conducted in a certified genetics laboratory. Briefly, DNA was extracted from this sample and genotyping was conducted using a real-time PCR system (polymerase chain reaction; Applied Biosystems^®^ Steponeplus^TM^ Real-time PCR system, USA). Internal controls, including reference samples (blank and negative controls), were used throughout, with contamination monitoring implemented at each step to ensure sample integrity. Positive controls for all genotypes were used from the Mexican branch of the CANDELA Consortium. Genotyping of the polymorphism *FTO* rs9939609 T>A (with three possible genotypes: TT, AT and AA) was conducted using a TaqMan single-nucleotide polymorphism (SNP) Genotyping Assay (Assay ID: C__30090620_10) and the reaction was performed in an Applied Biosystems 7500 Fast Real-Time PCR System (Applied Biosystems, Foster City, CA, USA). The results were analyzed using 7500 Software v2.0.5 (Applied Biosystems). Genomic DNA analyses that did not report a clear rs9939609 genotype were repeated. From the total, 30 samples were randomly selected and genotyped twice. We confirmed that the genotyping results perfectly agreed between duplicates.

Later, a complete anthropometric analysis was made by an experienced anthropometrist following the guidelines outlined by the International Society for the Advancement of Kinanthropometry (ISAK; [24]). The anthropometric measures included body mass and body height, skinfold thickness at six different locations (triceps, subscapular, abdominal, suprailium, thigh and mid-calf), body perimeters, bone diameters, heights and lengths [25].

For the second session, all the participants arrived at the laboratory in the morning (between 08:00 and 10:00), having followed the same diet and exercise standardizations for day 1. The day before each trial, participants were also required to refrain from consuming alcohol and caffeine and to maintain a sleeping pattern with at least 8 h of sleep the day before each trial. For this day, participants were encouraged to ingest 7 mL/kg of water two hours before arrival to increase the likelihood of euhydration. Upon arrival, participants voided and euhydration was confirmed by urine specific gravity < 1.020. Then, participants completed an incremental cycle ergometer (SNT Medical, Cardgirus, Barcelona, Spain) test to measure MFO and VO_2peak_. This test was preceded by a standardized warm-up (i.e., 10 min at 50 W for men, and 30 W for women). After the warm-up, the workload was increased by 25 W for men and 15 W for women every 3 min until the respiratory exchange ratio reached 1.0 (at this point, fat oxidation rate equals 0.0 (zero)). Thereafter, incremental loads were produced every minute until the participant reached volitional exhaustion, which happened after 3–7 stages. Participants were instructed to maintain a cycle cadence of 70 to 90 rpm during the whole test. The maximal exercise test finished when participants were unable to maintain a cycle cadence > 50 rpm or when they stopped pedaling due to fatigue. During the maximal exercise test, oxygen uptake (VO_2_) and carbon dioxide production (VCO_2_) were measured breath by breath by a gas analyzer (Metalyzer 3B, Cortex, Leipzig, Germany) which was calibrated before each measurement with certified gasses and a volume syringe. Substrate oxidation (i.e., carbohydrate and fat oxidation) rates were calculated at each stage with stoichiometric calculations [26]. For these calculations, VO_2_ and VCO_2_ data within the last 60 s of each 3 min period were used as a representative value. During exercise, heart rate and participants’ rating of perceived exertion (6–20 arbitrary units (a.u.), using the Borg scale [27], were recorded at each load. In this test, the intensity that produced the maximal rate of fat oxidation was registered as Fatmax. Last, VO_2peak_ was defined as the highest VO_2_ value obtained during the test. VO_2peak_ was considered more suitable as a definition of the variable measured in this test as criteria to consider the peak value as the maximal oxygen uptake (VO_2max_ (1); a rating of perceived exertion higher than 19 a.u. on the Borg scale, VO_2_ difference between the last two consecutive loads < 0.15 L/min, respiratory exchange ratio was higher than 1.10 and a heart rate greater than 80% of the age-adjusted estimate of maximal heart rate [28]) was not obtained in all participants.

### 2.5. Statistical Analysis

Initially, we determined whether the *FTO* rs9939609 genotype distribution in the sample met the Hardy–Weinberg equilibrium (HWE) by using a Chi-square test. A Chi-square test was also used to verify if the genotype frequency in our cohort was different from the 1000 Genome database of ethnically matched controls [29]. Specifically, the genotype distribution in our cohort was compared to that of the Caucasian/European population (TT, 37.2%; AT, 42.9%; and AA, 19.9.%). Categorical variables, such as the number of men and women in each genotype, were expressed as frequencies and tested by Chi-square tests. The normality of the continuous variables was tested using the Kolmogorov–Smirnov test. As all variables presented a normal distribution, data were presented as mean and standard deviation. Unpaired t-tests were used to compare the means in the descriptive variables according to sex. Initially, the whole sample was analyzed as a combined group, with men and women together, to enhance the statistical power of the analysis. A one-way analysis of variance (ANOVA) was used to compare data among the different *FTO* genotypes (TT vs. AT vs. AA). In the case of a significant F test (Geisser–Greenhouse correction for the assumption of sphericity), the Bonferroni post hoc analysis was employed to identify differences in pairwise comparison. When comparing the dominant (TT vs. A allele carriers) and recessive (AA vs. T allele carriers) models [30], the differences were calculated with unpaired *t*-tests. Additionally, we performed an analysis to assess the association of the *FTO* rs9939609 with MFO depending on the sex. A two-way ANOVA (2 × 3; sex × genotype) was used to compare data among the different *FTO* genotypes in the sub-samples of men and women. In the case of a significant sex × genotype interaction, the Bonferroni post hoc analysis was employed to identify differences in pairwise genotype comparison within each sex. The significance level was set at *p* < 0.050. All statistical analyses were performed using IBM SPSS statistics software 28.0.

## 3. Results

### 3.1. Whole Group

Genotyping success was 100%, with the following genotype distribution for the whole sample: TT, 40.0%; AT, 38.8%; and AA, 21.2% (Table 2). The distribution of the *FTO* genotypes was according to the Hardy–Weinberg equilibrium (*p* = 0.287). Additionally, the genotype distribution in the sample of participants was comparable with the Caucasian/European population of the 1000 Genome database (*p* = 0.780).

Table 2 presents all variables under investigation depending on the *FTO* rs9939609 genotype. Age, body characteristics and blood pressure variables were similar across all three genotypes (Table 2). Blood glucose, serum cholesterol and triglyceride concentrations and the levels of IL-6 and CPR were similar in all three genotypes. However, the serum insulin concentration was affected by the *FTO* rs9939609 genotype with higher values in AA with respect to AT and TT participants (*p* < 0.050). The HOMA-IR value was affected by the *FTO* rs9939609 genotype, with higher values in AA with respect to AT and TT participants (*p* < 0.050). Exercise variables, including VO_2peak_, MFO in absolute units or relativized by lean body mass, Fatmax or heart rate at Fatmax were similar across genotypes.

Table 3 depicts variables under investigation depending on the dominant (TT vs. A allele carriers) and recessive (AA vs. T allele carriers) models of the *FTO* rs9939609 polymorphism. In the dominant model, there was no statistical difference between TT and A allele carriers. However, the recessive model revealed that AA participants had higher values of body mass, body mass index, blood insulin concentration and HOMA-IR than T allele carriers (*p* < 0.050). Still, there were no differences in fat oxidation variables during exercise when comparing genotype variants in the dominant or recessive models (Table 3).

### 3.2. Sex × Genotype Interaction

**Figure 1:**
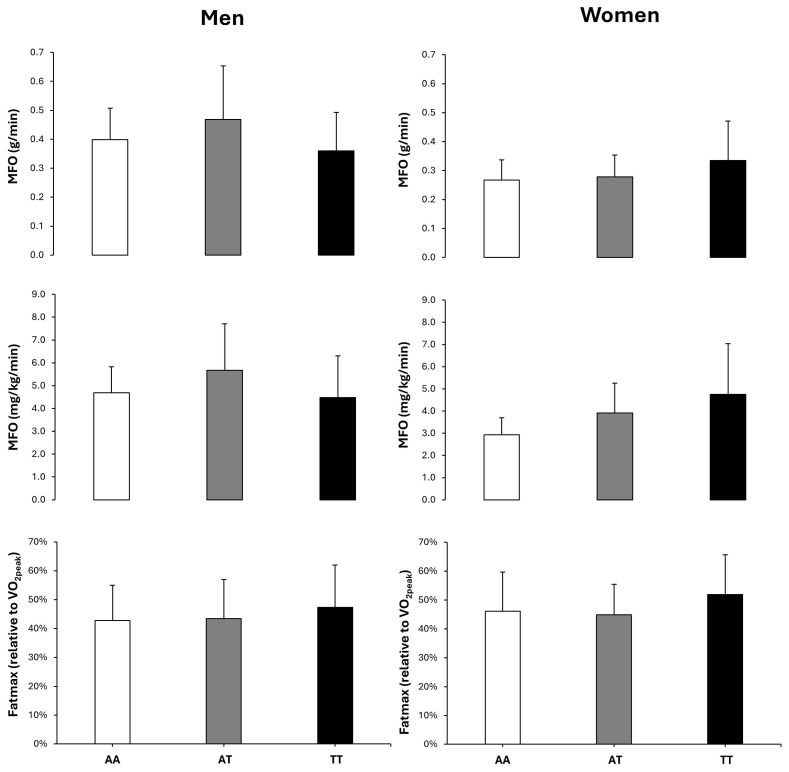
Maximal fat oxidation (MFO) rate in absolute units or relativized by lean body mass and exercise intensity that elicited MFO (Fatmax) obtained during a ramp exercise test in healthy men and women according to their genotype of *FTO* rs9939609. Data are mean (standard deviation). A two-way ANOVA (2 × 3, sex × genotype) was used to detect the main effect of sex and genotype and their interaction (see Table 4 for details of the statistical analysis of this section).

Table 4 depicts the main effects of sex, genotype and their interaction on the variables under investigation. There was a main effect of sex for the height, body fat, systolic blood pressure, serum HDL and CRP concentrations, VO_2peak_, MFO, MFO/lean body mass and Fatmax. In all these variables, the values of men were higher than in women (*p* < 0.050), except for body fat and serum HDL concentration, where data in women were higher than in men (*p* < 0.050). There was a main effect of genotype for body fat, serum insulin concentration and HOMA-IR, with values always higher in AA vs. AT and TT (*p* < 0.050). The post hoc analysis revealed that these differences in body fat, serum insulin concentration and HOMA-IR were present in both men and women (*p* < 0.050). However, there was no sex × genotype interaction in any of the variables under investigation. Exercise variables, including VO_2peak_, MFO in absolute units or relativized by lean body mass, Fatmax or heart rate at Fatmax were similar across genotypes in both men and women (Figure 1). Overall, the two-way ANOVA including sex × genotype interactions reflected similar differences among/between genotypes that were found in the one-way ANOVA of the whole group (i.e., men and women together in the same group).

## 4. Discussion

The aim of this study was to explore the association between the *FTO* rs9939609 polymorphism and MFO and Fatmax during exercise in healthy individuals. This may be important to ascertain a potential genetic predisposition to obesity as the ability to use fat as fuel during exercise has been linked to obesity and insulin resistance. The main outcomes of this investigation indicated the following: (a) While participants with different *FTO* rs9939609 genotypes had comparable age, body characteristics, VO_2peak_, blood pressure and blood variables, fasting serum insulin concentration and HOMA-IR were higher in AA with respect to AT and TT participants. This indicates that despite similar characteristics and aerobic fitness, AA participants were categorized as having a higher tendency to develop insulin resistance [31]. (b) Despite the fact that AA participants tended to have higher body mass, BMI and aforementioned insulin resistance, their MFO values were similar to AT and TT participants, either in absolute units (g/min) or relative to lean body mass values (mg/kg/min), with comparable Fatmax values. The lack of association of the *FTO* rs9939609 polymorphism with fat oxidation during exercise was independent of participants’ sex, as MFO was comparable across genotypes in the subsamples of men and women (Figure 1). This suggests that AA participants (either men or women) did not possess an inferior ability to oxidize fat during exercise, while they presented several obesity-related phenotypes. (c) The recessive model that compares T allele carriers (TT and AT) to AA homozygotes was the best model to categorize the effect of the *FTO* rs9939609 polymorphism in metabolic syndrome-related variables, as AA participants had higher values of body mass, body mass index, blood insulin concentration and HOMA-IR than T allele carriers. Collectively, all this information suggests that the *FTO* rs9939609 polymorphism was associated with several phenotypes associated with obesity and insulin resistance, particularly under the AA vs. T allele/recessive model. However, the *FTO* rs9939609 polymorphism was not associated with MFO or Fatmax either when comparing all three genotypes or under the dominant or recessive models. This suggests that reduced fat oxidation during exercise is not a cause of the obesogenic effect of the *FTO* AA genotype, at least in healthy individuals.

The association between the *FTO* rs9939609 polymorphism and fat oxidation rate during exercise has recently been tested by Ponce-Gonzalez et al. [11] in young adults (men and women). These authors reported that MFO values were higher in the TT participants compared to the AT group, in addition to higher values of hunger and appetite after exercise. These differences were present despite AT participants having comparable obesity-related factors including dietary intake, physical activity expenditure, body composition and resting metabolic rate and cardiorespiratory fitness than TT. Additionally, individuals with the AA genotype exhibited comparable MFO values to those with the TT genotype, despite AA genotypes being habitually associated with higher BMI and fat mass compared to AT and TT [11]. Our investigation followed a similar design to Ponce-Gonzalez et al. (2023) as we recruited adults (men and women) with no diagnosed condition, and we tested the effect of the *FTO* rs9939609 polymorphism in participants undergoing a maximal cycle ergometer test with 3 min stages to assess MFO. However, we failed to demonstrate any effect of the *FTO* rs9939609 polymorphism on MFO and Fatmax, despite similarities in the protocols. In the Ponce-Gonzalez et al. (2023) study, lower MFO values in AT participants were obtained with similar Fatmax values. Additionally, in their study, AT participants did not have higher body mass or fat mass, with similar blood variables to the other genotypes, suggesting that the potential lower fat oxidation of AT individuals was not associated with any clinical consequence. Interestingly, in our study, participants with the AA genotype of the *FTO* rs9939609 polymorphism exhibited several signs associated with obesity (Table 3). Specifically, AA individuals had statistically significant higher values of body mass, BMI, fasting insulin concentration and HOMA-IR compared to T allele carriers, in line with outcomes observed in the previous literature [32]. Additionally, although not statistically significant, AA participants showed higher values of body fat percentage, diastolic blood pressure, fasting blood glucose concentration and serum triglyceride concentration, accompanied with lower levels of HDL concentration. These findings suggest that, despite being classified as healthy, our sample of *FTO* AA participants may be in a preclinical stage of developing metabolic syndrome and insulin resistance, particularly when compared to T allele carriers. The elevated fasting insulin concentrations and HOMA-IR values observed in AA individuals could pose a significant physiological burden, even among young and ostensibly healthy individuals, as these markers are predictive of future glucose homeostasis dysregulation and an increased risk of type 2 diabetes. Importantly, even among these individuals who had already developed some obesity-related traits, the *FTO* rs9939609 polymorphism was not associated with any statistically significant effect on fat oxidation during exercise. This represents a novel finding in the literature, challenging the assumption that the *FTO* gene directly influences exercise-related metabolic pathways.

The A allele of the *FTO* rs9939609 polymorphism has also been associated with increased energy intake and higher dietary carbohydrate and fat intake [33]. Additionally, the A allele has been associated with increased hunger/lowered satiety but is not associated with altered resting energy expenditure or low physical activity in humans [15]. This could be explained because the protein that is encoded by the *FTO* gene is highly expressed in the hypothalamus region involved in the regulation of food intake [34]. Specifically, the FTO protein acts as an m6A demethylase, removing methyl groups from N6-methyladenosine (m6A) residues on RNA molecules. This demethylation process affects various aspects of RNA function, including splicing, stability, translation and degradation, thereby impacting the expression of genes involved in metabolic and cellular processes [35]. The *FTO* AA genotype produces a higher expression of FTO protein, as the A allele is thought to affect regulatory regions of the *FTO* gene, leading to enhanced transcriptional activity [36]. Higher levels of FTO protein are associated with increased hunger, food intake and preference for high-calorie foods; reduced satiety; and, ultimately, alterations in body fat storage [15]. Therefore, the interaction between different *FTO* genotypes and obesity-related factors may primarily operate through the influence of this genetic variant on eating behaviors and food cravings, rather than directly affecting fat oxidation [37]. Although the development of obesity may be partially influenced by genetically determined lower levels of fat oxidation, our study found no significant association between the genotypes of the *FTO* rs9939609 polymorphism and fat oxidation rates during exercise. This aligns with findings from other studies that similarly reported no significant relationship between *FTO* genotypes and fat metabolism during fasting or postprandial states at rest [10]. It is reasonable to assume that genetics, or epigenetics, may contribute to fat oxidation rate at rest and during exercise, but it appears that the *FTO* rs9939609 polymorphism has only a marginal contribution to these phenotypes.

Research has demonstrated that an appropriate level of physical activity may overcome some of the adverse effects of the *FTO* rs9939609 variant on occurring obesity [38]. Indeed, the association of the *FTO* A-risk allele with the odds of obesity is attenuated by 27% in physically active adults [39]. Studies on the interaction between the *FTO* gene and environmental factors suggest that the impact of this genotype on obesity can be significantly influenced by lifestyle choices. These findings emphasize the importance of lifestyle interventions, such as diet and physical activity, in mitigating obesity risk among individuals who are genetically predisposed [40]. Clinically, these findings underscore the necessity of personalized interventions that account for genetic predisposition to effectively manage and reduce the risk of obesity and its associated comorbidities. Emphasis should be placed on implementing diet and exercise interventions as foundational pillars for achieving sustainable, long-term health improvements.

This study has several limitations that warrant consideration. First, the modest sample size of 80 healthy participants (41 men and 39 women) may limit the generalizability of our findings and the statistical power to detect subtle genetic associations, particularly in subgroup analyses. While the sample size was sufficient to detect significant differences among genotypes for some obesity-related phenotypes, larger cohorts are needed to confirm these findings and explore additional interactions. Second, our study focused exclusively on healthy individuals, which restricts the applicability of the results to populations with different metabolic profiles, such as individuals with obesity or overweight. The metabolic and physiological responses to the *FTO* rs9939609 polymorphism observed in this healthy cohort may differ significantly from those in populations with existing metabolic syndrome or other health conditions. Future research should extend these investigations to include a broader spectrum of participants with varying health and body composition statuses. Third, we examined only a single-nucleotide polymorphism (SNP) of the *FTO* gene. It is possible that other SNPs within the *FTO* gene, such as rs1421085 [41] and rs17817449 [42], may also have influence as they have been associated with interindividual variations in obesity and appetite control. Within the context of genetic predisposition to obesity, multiple genes and their respective SNPs may contribute to obesity risk, potentially exerting cumulative effects wherein the presence of “negative” alleles increases susceptibility in an additive manner. Consequently, it is essential to investigate the interplay of these genetic variants to assess their association with fat oxidation during exercise in future investigations. Another important limitation of this study is that we did not control participants’ dietary patterns or physical activity levels. While this decision was made to avoid influencing or altering the participants’ normal behaviors, it introduces the possibility that variations in diet or physical activity could have impacted the results of the study. Finally, our cross-sectional study design precludes any causal interpretation of the observed associations. Longitudinal studies are needed to elucidate the temporal dynamics of how the *FTO* rs9939609 polymorphism and related genetic factors influence fat oxidation and obesity-related phenotypes over time.

## 5. Conclusions

In summary, the *FTO* rs9939609 polymorphism was associated with several phenotypes associated with obesity and insulin resistance such as increased body mass, body mass index and higher fasting insulin concentration and HOMA-IR in otherwise healthy participants, particularly under the recessive model for this genetic variation (AA vs. T allele carriers). This demonstrates that possessing two copies of the *FTO* A allele (i.e., AA genotype) is associated with a higher predisposition to the development of obesity-related phenotypes. However, the *FTO* rs9939609 polymorphism was not associated with MFO during exercise as fat oxidation variables were comparable in participants with the AA, AT or TT allele types. Overall, the association of the *FTO* AA genotype with higher fasting insulin concentration and HOMA-IR was observed similarly in both men and women, despite notable sex-related differences in anthropometric and exercise fitness variables. These findings suggest that the potential negative association of the *FTO* polymorphism with obesity-related phenotypes is evident in both healthy men and women. However, the *FTO* AA genotype did not appear to impair fat oxidation during exercise in either sex. This suggests that reduced fat oxidation during exercise may not be a cause of the obesogenic-like effect of the *FTO* AA genotype, at least in healthy individuals.

## Figures and Tables

**Table 1:** Participants’ age, morphological characteristics and maximal values at the end of a maximal ramp test on a cycle ergometer. For the whole sample (*n* = 80), and for men (*n* = 41) and women (*n* = 39).

Variable (Units)	All	Men	Women	*p* Value
Age (years)	33.36 (7.67)	33.29 (7.68)	33.44 (7.75)	0.939
Body mass (kg)	81.81 (16.71)	85.22 (14.05)	78.23 (18.62)	0.194
Height (cm)	170.11 (8.57)	179.06 (6.65)	163.82 (5.20) *	<0.001
Body mass index (kg/m^2^)	28.37 (6.12)	27.53 (4.74)	29.25 (7.26)	0.476
Body fat (%)	20.16 (9.23)	14.02 (5.43)	26.61 (7.92) *	<0.001
Systolic blood pressure (mmHg)	114 (13)	119 (12)	109 (12)	0.235
Diastolic blood pressure (mmHg)	75 (10)	75 (10)	76 (10)	0.824
Mean arterial blood pressure (mmHg)	88.20 (10.11)	89.47 (9.92)	86.87 (10.26)	0.721
Blood glucose concentration (mg/dL)	87.43 (10.52)	88.40 (11.22)	86.44 (9.79)	0.558
Serum HDL concentration (mg/dL)	51.96 (12.79)	48.70 (11.82)	55.31 (13.03) *	0.030
Serum LDL concentration (mg/dL)	102.31 (29.86)	101.08 (30.23)	103.54 (29.83)	0.755
Serum triglycerides concentration (mg/dL)	89.69 (57.23)	98.26 (65.56)	81.13 (46.77)	0.905
VO_2peak_ (mL/kg/min)	32.49 (10.83)	37.62 (10.15)	27.09 (8.78) *	<0.001
Peak heart rate (beat/min)	186 (6)	186 (6)	186 (6)	0.772

Data are mean (standard deviation). * depicts a statistically significant difference between men and women at *p* < 0.050 analyzed with an unpaired *t* test. VO_2peak_ = peak oxygen uptake.

**Table 2:** Age, body characteristics, blood pressure, blood variables and fat oxidation variables during exercise in healthy participants according to their genotype of the *FTO* rs9939609.

Variable (Units)	TT	AT	AA	*p* Value
Men/women (number)	17/15	15/16	9/8	0.920
Age (years)	32.56 (7.34)	33.06 (7.34)	35.41 (8.23)	0.453
Body mass (kg)	78.71 (17.06)	81.02 (16.23)	89.07 (15.57)	0.111
Height (cm)	170.78 (7.55)	169.44 (9.41)	170.02 (9.18)	0.827
Body mass index (kg/m^2^)	27.07 (6.20)	28.19 (5.30)	31.16 (6.82)	0.081
Body Fat (%)	18.30 (8.58)	20.45 (8.60)	23.12 (11.08)	0.216
Systolic blood pressure (mmHg)	113 (13)	116 (12)	114 (13)	0.681
Diastolic blood pressure (mmHg)	73 (8)	76 (10)	78 (13)	0.199
Mean arterial blood pressure (mmHg)	86.30 (8.45)	89.17 (10.53)	90.04 (12.08)	0.376
Blood glucose concentration (mg/dL)	85.29 (6.58)	87.65 (14.14)	90.94 (7.81)	0.205
Serum HDL concentration (mg/dL)	54.29 (13.18)	50.90 (12.62)	49.65 (12.43)	0.413
Serum LDL concentration (mg/dL)	106.19 (33.87)	97.93 (24.63)	102.94 (31.24)	0.561
Serum triglycerides concentration (mg/dL)	77.29 (29.69)	99.10 (79.30)	95.71 (47.01)	0.297
Serum CRP concentration (mg/dL)	3.16 (4.94)	2.50 (3.31)	2.97 (3.48)	0.807
Serum IL-6 concentration (pg/mL)	2.03 (1.41)	2.26 (2.33)	2.61 (2.02)	0.622
Serum insulin concentration (μU/mL)	8.62 (6.91) *	7.19 (4.18) *	13.45 (10.77)	0.015
HOMA-IR	1.85 (1.60) *	1.58 (0.98) *	3.16 (2.82)	0.010
VO_2peak_ (mL/kg/min)	31.99 (8.92)	33.22 (10.68)	32.08 (14.46)	0.893
MFO (g/min)	0.35 (0.13)	0.37 (0.11)	0.33 (0.11)	0.702
MFO/lean body mass (mg/kg/min)	4.61 (2.01)	4.73 (1.90)	3.85 (1.31)	0.275
Fatmax (%VO_2peak_)	49.47 (14.23)	44.16 (11.94)	44.19 (12.54)	0.210
Heart rate at Fatmax	110 (17)	107 (17)	111 (15)	0.707

Data are mean (standard deviation). A one-way ANOVA was used to compare data among genotypes (TT vs. AT vs. AA) and the Bonferroni post hoc analysis was employed to identify differences in pairwise comparison. * depicts a statistically significant difference from AA participants at *p* < 0.050. MFO = maximal fat oxidation rate. VO_2peak_ = peak oxygen uptake. HOMA-IR = homeostatic model assessment for insulin resistance.

**Table 3:** Age, body characteristics, blood pressure, blood variables and fat oxidation variables during exercise in healthy participants according to their genotype of the *FTO* rs9939609 using the dominant (TT vs. A allele carriers) and recessive (AA vs. T allele carriers) models.

	Dominant Model			Recessive Model		
Variable (Units)	TT	A Allele	*p* Value	T Allele	AA	*p* Value
Men/women (number)	17/15	24/24	0.784	32/31	9/8	0.875
Age (years)	32.56 (7.34)	33.90 (7.91)	0.449	32.81 (7.48)	35.41 (8.23)	0.216
Body mass (kg)	78.71 (17.06)	83.88 (16.31)	0.177	79.85 (16.57)	89.07 * (15.57)	0.042
Height (cm)	170.78 (7.55)	169.65 (9.24)	0.565	170.13 (8.48)	170.02 (9.18)	0.967
Body mass index (kg/m^2^)	27.07 (6.20)	29.24 (5.99)	0.121	27.62 (5.75)	31.16 (6.82)	0.033
Body Fat (%)	18.30 (8.58)	21.40 (9.52)	0.142	19.36 (8.59)	23.12 (11.08)	0.136
Systolic blood pressure (mmHg)	113 (13)	115 (15)	0.478	115 (13)	114 (13)	0.865
Diastolic blood pressure (mmHg)	73 (8)	77 (11)	0.103	75 (9)	78 (13)	0.166
Mean arterial blood pressure (mmHg)	86.30 (8.45)	89.48 (10.98)	0.169	87.71 (9.56)	90.04 (12.08)	0.403
Blood glucose concentration (mg/dL)	85.29 (6.58)	88.81 (14.14)	0.147	86.47 (11.00)	90.94 (7.81)	0.121
Serum HDL concentration (mmol/L)	54.29 (13.18)	50.46 (12.44)	0.195	52.60 (12.91)	49.65 (12.43)	0.403
Serum LDL concentration (mmol/L)	106.19 (33.87)	99.74 (26.98)	0.354	102.13 (29.73)	102.94 (31.24)	0.921
Serum triglycerides concentration (mmol/L)	77.29 (29.69)	97.87 (68.82)	0.120	88.02 (60.01)	95.71 (47.01)	0.627
Blood CRP concentration (mg/dL)	3.16 (4.94)	2.66 (3.35)	0.595	2.83 (4.19)	2.97 (3.48)	0.898
Blood IL-6 concentration (pg/mL)	2.03 (1.41)	2.39 (2.21)	0.392	2.13 (1.91)	2.61 (2.02)	0.360
Blood insulin concentration (μU/mL)	8.62 (6.91)	9.41 (7.74)	0.645	7.91 (5.71)	13.45 (10.77) *	0.005
HOMA-IR	1.85 (1.60)	2.14 (1.97)	0.501	1.72 (1.32)	3.16 (2.82) *	0.003
VO_2peak_ (mL/kg/min)	31.99 (8.92)	32.82 (12.01)	0.740	32.60 (9.77)	32.08 (14.46)	0.863
MFO (g/min)	0.35 (0.13)	0.36 (0.15)	0.747	0.36 (0.15)	0.33 (0.11)	0.569
MFO/lean body mass (mg/kg/min)	4.61 (2.01)	4.42 (1.76)	0.649	4.67 (1.95)	3.85 (1.31)	0.111
Fatmax (%VO_2peak_)	49.47 (14.23)	44.18 (12.01)	0.070	46.86 (13.31)	44.19 (12.54)	0.471
Heart rate at Fatmax	110 (17)	109 (16)	0.706	109 (17)	111 (15)	0.601

Data are mean (standard deviation). When comparing the dominant (TT vs. A allele carriers) and recessive (AA vs. T allele carriers) models, the differences were calculated with unpaired *t*-tests. * depicts a statistically significant difference between AA homozygotes and T allele participants at *p* < 0.050. MFO = maximal fat oxidation rate. VO_2peak_ = peak oxygen uptake. HOMA-IR = homeostatic model assessment for insulin resistance.

**Table 4:** Sex × genotype interaction in healthy men and women according to their genotype of the *FTO* rs9939609.

Variable (Units)	Sex	Genotype	Interaction
Age (years)	0.982	0.450	0.425
Body mass (kg)	0.189	0.083	0.154
Height (cm)	<0.001	0.837	0.492
Body mass index (kg/m^2^)	0.055	0.053	0.064
Body Fat (%)	<0.001	0.039	0.091
Systolic blood pressure (mmHg)	0.002	0.538	0.578
Diastolic blood pressure (mmHg)	0.508	0.190	0.625
Mean arterial blood pressure (mmHg)	0.398	0.340	0.594
Blood glucose concentration (mg/dL)	0.507	0.206	0.273
Serum HDL concentration (mg/dL)	0.013	0.398	0.159
Serum LDL concentration (mg/dL)	0.722	0.570	0.537
Serum triglycerides concentration (mg/dL)	0.207	0.253	0.186
Serum CRP concentration (mg/dL)	0.019	0.756	0.360
Serum IL-6 concentration (pg/mL)	0.447	0.602	0.499
Serum insulin concentration (μU/mL)	0.932	0.018	0.999
HOMA-IR	0.952	0.014	0.996
VO_2peak_ (mL/kg/min)	<0.001	0.730	0.054
MFO (g/min)	<0.001	0.538	0.312
MFO/lean body mass (mg/kg/min)	0.010	0.170	0.100
Fatmax (%VO_2peak_)	<0.001	0.890	0.268
Heart rate at Fatmax	0.645	0.699	0.601

Data represent the p value for each main effect obtained through a two-way ANOVA (2 × 3, sex × genotype). MFO = maximal fat oxidation rate. VO_2peak_ = peak oxygen uptake. HOMA-IR = homeostatic model assessment for insulin resistance.

## Data Availability

The data presented in this study are available on request from the corresponding authors due to restrictions associated imposed by the funder of this investigation.

## References

[1] The Challenge of Obesity. https://www.who.int/europe/news-room/fact-sheets/item/the-challenge-of-obesity?utm_source=chatgpt.com.

[2] Bozkurt B., Aguilar D., Deswal A., Dunbar S.B., Francis G.S., Horwich T., Jessup M., Kosiborod M., Pritchett A.M., Ramasubbu K. (2016). Contributory Risk and Management of Comorbidities of Hypertension, Obesity, Diabetes Mellitus, Hyperlipidemia, and Metabolic Syndrome in Chronic Heart Failure: A Scientific Statement from the American Heart Association. Circulation.

[3] Petkeviciene J., Smalinskiene A., Klumbiene J., Petkevicius V., Kriaucioniene V., Lesauskaite V. (2016). Physical Activity, but Not Dietary Intake, Attenuates the Effect of the FTO Rs9939609 Polymorphism on Obesity and Metabolic Syndrome in Lithuanian Adult Population. Public Health.

[4] Archer E., Lavie C.J., Hill J.O. (2018). The Contributions of “Diet”, “Genes”, and Physical Activity to the Etiology of Obesity: Contrary Evidence and Consilience. Prog。 Cardiovasc. Dis..

[5] Schutz Y., Tremblay A., Weinsier R.L., Nelson K.M. (1992). Role of Fat Oxidation in the Long-Term Stabilization of Body Weight in Obese Women. Am. J. Clin. Nutr..

[6] Ranneries C., Bülow J., Buemann B., Christensen N.J., Madsen J., Astrup A. (1998). Fat Metabolism in Formerly Obese Women. Am. J. Physiol. Endocrinol. Metab..

[7] Lanzi S., Codecasa F., Cornacchia M., Maestrini S., Salvadori A., Brunani A., Malatesta D. (2014). Fat Oxidation, Hormonal and Plasma Metabolite Kinetics during a Submaximal Incremental Test in Lean and Obese Adults. PLoS ONE.

[8] Maunder E., Plews D.J., Kilding A.E. (2018). Contextualising Maximal Fat Oxidation During Exercise: Determinants and Normative Values. Front. Physiol..

[9] Goodpaster B.H., Wolfe R.R., Kelley D.E. (2002). Effects of Obesity on Substrate Utilization during Exercise. Obes. Res..

[10] Corpeleijn E., Petersen L., Holst C., Saris W.H., Astrup A., Langin D., MacDonald I., Martinez J.A., Oppert J.M., Polak J. (2010). Obesity-Related Polymorphisms and Their Associations with the Ability to Regulate Fat Oxidation in Obese Europeans: The NUGENOB Study. Obesity.

[11] Ponce-Gonzalez J.G., Martínez-Ávila Á., Velázquez-Díaz D., Perez-Bey A., Gómez-Gallego F., Marín-Galindo A., Corral-Pérez J., Casals C. (2023). Impact of the FTO Gene Variation on Appetite and Fat Oxidation in Young Adults. Nutrients.

[12] Scuteri A., Sanna S., Chen W.M., Uda M., Albai G., Strait J., Najjar S., Nagaraja R., Orrú M., Usala G. (2007). Genome-Wide Association Scan Shows Genetic Variants in the FTO Gene Are Associated with Obesity-Related Traits. PLoS Genet..

[13] Peng S., Zhu Y., Xu F., Ren X., Li X., Lai M. (2011). FTO Gene Polymorphisms and Obesity Risk: A Meta-Analysis. BMC Med..

[14] Frayling T.M., Timpson N.J., Weedon M.N., Zeggini E., Freathy R.M., Lindgren C.M., Perry J.R.B., Elliott K.S., Lango H., Rayner N.W. (2007). A Common Variant in the FTO Gene Is Associated with Body Mass Index and Predisposes to Childhood and Adult Obesity. Science.

[15] Speakman J.R. (2015). The “Fat Mass and Obesity Related” (FTO) Gene: Mechanisms of Impact on Obesity and Energy Balance. Curr. Obes. Rep..

[16] Graff M., Scott R.A., Justice A.E., Young K.L., Feitosa M.F., Barata L., Winkler T.W., Chu A.Y., Mahajan A., Hadley D. (2017). Genome-Wide Physical Activity Interactions in Adiposity—A Meta-Analysis of 200,452 Adults. PLoS Genet..

[17] Montes-de-Oca-García A., Perez-Bey A., Corral-Pérez J., Velázquez-Díaz D., Opazo-Díaz E., Fernandez-Santos J.R., Rebollo-Ramos M., Amaro-Gahete F.J., Cuenca-García M., Ponce-González J.G. (2021). Maximal Fat Oxidation Capacity Is Associated with Cardiometabolic Risk Factors in Healthy Young Adults. Eur. J. Sport Sci..

[18] Warburton D.E.R., Bredin S.S.D., Jamnik V.K., Gledhill N. (2011). Validation of the PAR-Q+ and EPARmed-X+. Health Fit. J. Can..

[19] Ruíz-Moreno C., Gutiérrez-Hellín J., González-García J., GiráLdez-Costas V., Brito de Souza D., Del Coso J. (2021). Effect of Ambient Temperature on Fat Oxidation during an Incremental Cycling Exercise Test. Eur. J. Sport Sci..

[20] Muñoz A., Aguilar-Navarro M., Ruiz-Moreno C., Varillas-Delgado D., Amaro-Gahete F.J., Gutiérrez-Hellín J., Del Coso J., López-Samanes Á. (2024). Influence of the Time of Day in the Effect of Caffeine on Maximal Fat Oxidation during Exercise in Women: A Randomized, Crossover, Double-Blind, and Placebo-Controlled Study. Eur. J Appl. Physiol..

[21] Papaioannou T.G., Protogerou A.D., Vrachatis D., Konstantonis G., Aissopou E., Argyris A., Nasothimiou E., Gialafos E.J., Karamanou M., Tousoulis D. (2016). Mean Arterial Pressure Values Calculated Using Seven Different Methods and Their Associations with Target Organ Deterioration in a Single-Center Study of 1878 Individuals. Hypertens. Res..

[22] Matthews D.R., Hosker J.P., Rudenski A.S., Naylor B.A., Treacher D.F., Turner R.C. (1985). Homeostasis Model Assessment: Insulin Resistance and Beta-Cell Function from Fasting Plasma Glucose and Insulin Concentrations in Man. Diabetologia.

[23] Sambrook J., Russell D.W. (2001). Molecular Cloning: A Laboratory Manual.

[24] Marfell-Jones M., Olds T., Stewart A., Carter J. (2006). International Standards for Anthropometric Assessment.

[25] Carter J.E.L. (2002). Part 1: The Heath-Carter Anthropometric Somatotype-Instruction Manual.

[26] Brouwer E. (1957). On Simple Formulae for Calculating the Heat Expenditure and the Quantities of Carbohydrate and Fat Oxidized in Metabolism of Men and Animals, from Gaseous. Acta Physiol. Pharmacol. Neerl..

[27] Borg G. (1990). Psychophysical Scaling with Applications in Physical Work and the Perception of Exertion *Scand*. J. Work Environ. Health.

[28] Edvardsen E., Hem E., Anderssen S.A. (2014). End Criteria for Reaching Maximal Oxygen Uptake Must Be Strict and Adjusted to Sex and Age: A Cross-Sectional Study. PLoS ONE.

[29] rs9939609 (SNP)—Population Genetics—Homo_sapiens—Ensembl Genome Browser 113. https://www.ensembl.org/Homo_sapiens/Variation/Phenotype?r=16:53786115-53787115;v=rs9939609;vdb=variation;vf=923521178.

[30] Prakash J., Mittal B., Srivastava A., Awasthi S., Srivastava N. (2016). Association of FTO Rs9939609 SNP with Obesity and Obesity- Associated Phenotypes in a North Indian Population. Oman. Med. J..

[31] Gayoso-Diz P., Otero-González A., Rodriguez-Alvarez M.X., Gude F., García F., De Francisco A., Quintela A.G. (2013). Insulin Resistance (HOMA-IR) Cut-off Values and the Metabolic Syndrome in a General Adult Population: Effect of Gender and Age: EPIRCE Cross-Sectional Study. BMC Endocr. Disord..

[32] Liguori R., Labruna G., Alfieri A., Martone D., Farinaro E., Contaldo F., Sacchetti L., Pasanisi F., Buono P. (2014). The FTO Gene Polymorphism (Rs9939609) Is Associated with Metabolic Syndrome in Morbidly Obese Subjects from Southern Italy. Mol. Cell. Probes..

[33] Sonestedt E., Roos C., Gullberg B., Ericson U., Wirfält E., Orho-Melander M. (2009). Fat and Carbohydrate Intake Modify the Association between Genetic Variation in the FTO Genotype and Obesity. Am. J. Clin. Nutr..

[34] Hofker M., Wijmenga C. (2009). A Supersized List of Obesity Genes. Nat. Genet..

[35] Gao Z., Zha X., Li M., Xia X., Wang S. (2024). Insights into the M6A Demethylases FTO and ALKBH5: Structural, Biological Function, and Inhibitor Development. Cell Biosci..

[36] Yin D., Li Y., Liao X., Tian D., Xu Y., Zhou C., Liu J., Li S., Zhou J., Nie Y. (2023). FTO: A Critical Role in Obesity and Obesity-Related Diseases. Br. J. Nutr..

[37] Abdella H.M., Farssi H.O.E., Broom D.R., Hadden D.A., Dalton C.F. (2019). Eating Behaviours and Food Cravings; Influence of Age, Sex, BMI and FTO Genotype. Nutrients.

[38] Ruiz J.R., Labayen I., Ortega F.B., Legry V., Moreno L.A., Dallongeville J., Martínez-Gómez D., Bokor S., Manios Y., Ciarapica D. (2010). Attenuation of the Effect of the FTO Rs9939609 Polymorphism on Total and Central Body Fat by Physical Activity in Adolescents: The HELENA Study. Arch. Pediatr. Adolesc. Med..

[39] Kilpeläinen T.O., Qi L., Brage S., Sharp S.J., Sonestedt E., Demerath E., Ahmad T., Mora S., Kaakinen M., Sandholt C.H. (2011). Physical Activity Attenuates the Influence of FTO Variants on Obesity Risk: A Meta-Analysis of 218,166 Adults and 19,268 Children. PLoS Med..

[40] Kalantari N., Doaei S., Keshavarz-Mohammadi N., Gholamalizadeh M., Pazan N. (2016). Review of Studies on the Fat Mass and Obesity-Associated (FTO) Gene Interactions with Environmental Factors Affecting on Obesity and Its Impact on Lifestyle Interventions. ARYA Atheroscler..

[41] Claussnitzer M., Dankel S.N., Kim K.-H., Quon G., Meuleman W., Haugen C., Glunk V., Sousa I.S., Beaudry J.L., Puviindran V. (2015). FTO Obesity Variant Circuitry and Adipocyte Browning in Humans. N. Engl. J. Med..

[42] Loos R.J.F., Yeo G.S.H. (2014). The Bigger Picture of FTO: The First GWAS-Identified Obesity Gene. Nat. Rev. Endocrinol..

